# Editorial: Papillomaviruses, immunity, and tumour development

**DOI:** 10.3389/fcimb.2025.1599910

**Published:** 2025-04-08

**Authors:** Simon Beddows, Hanna Kann, Filipe Colaço Mariz

**Affiliations:** ^1^ Virus Reference Department, Public Health Microbiology Division, UK Health Security Agency, London, United Kingdom; ^2^ Department of Microbiology and Immunology, Institute of Biomedicine, University of Gothenburg, Gothenburg, Sweden; ^3^ Tummorvirus-Specific Vaccination Strategies, German Cancer Research Center (DKFZ), Heidelberg, Baden-Württemberg, Germany

**Keywords:** papillomaviruses, HPV, immunity, tumour development, infection

The last decades have provided remarkable advances on the fight against HPV related diseases. Nevertheless, the scientific community remains engaged in advancing the current understanding of mechanisms driving HPV pathogenesis and of key determinants of the HPV vaccine effectiveness, which will assist with meeting the World Health Organization’s Global Strategy for cervical cancer elimination. In this Research Topic, we aimed to address the diversity and complexity of the interplay between the three elements - HPV, immunity and cancer - and in particular highlight the novelties and advances in the field, not least bringing into the spotlight topics that may be underrepresented in papillomavirus research.

Here, two works provide novel mechanistic evidence of host epithelium adaptation to high-risk HPV persistence and modulation of host immune responses by the beta-genus HPV E6 protein in the inherited skin disorder epidermodysplasia verruciformis (EV).

Gallego et al. employed a 3D cell culture model of HPV18-replicating stratified squamous epithelium (so far, the only experimental model of this type for studying productive HPV infection) to report evidence of reprogrammed expression and spatial distribution of connexins - a family of proteins that ensures integrity and function of Gap junction intercellular communication. Researchers found that the two isoforms of connexin played an important role in accelerating intercellular communication, which may be linked to epithelial adaptation and host immune surveillance. It will be important to determine if connexin modulation during viral infection is driven by HPV-specific replication machinery or rather the cell response to infection and the impact of both virus and cell genetic variability on this mechanism.

In individuals suffering from EV, cutaneous infection by HPV8 is commonly accompanied by non-melanoma skin cancer (NMSC) in sun-exposed areas. While many of the cellular mechanisms altered by HPV8 infection are unclear, Vella et al. have shed some light on the intriguing molecular networking orchestrated by HPV8 E6. Using RNAi-mediated knockdown and overexpression in organotypic air-liquid interface cultures, the authors demonstrated that HPV8 E6 oncoprotein drives a proinflammatory microenvironment via induction of monocyte-attracting chemokine CCL2 and downregulation of the recently identified tumour suppressor pathway C/EBPalfa/miR. In association with the immune suppressive role of HPV8 E7, the E6 may fuel carcinogenesis in EV lesions through chronic inflammation. It remains to be studied whether this feature is conserved among other beta HPV types and whether this could trigger chronic inflammation upon immunosuppression.

Alternative splicing events in the HPV genome add yet another layer of complexity to the host-pathogen interactions. In their recent review, Wang et al. have summarized knowledge on the spectrum of splicing isoforms, their regulation and function, and hypothesize that comparing splicing patterns across the HPV types could at least partly explain the differences in their oncogenic potential.

As some molecular mechanisms affect the interplay between virus and host, they also become suspected targets for biomarkers or even for therapies. Here, Albano et al. report that i) a particular type of post-translational modification, citrullination, plays a role in cervical cancer pathogenesis; ii) levels of peptidyl-arginine deiminase 4 (PAD4) may be a tool for assessing disease progression and iii) PAD inhibitor, BB-Cl-A, may be a promising drug candidate. In contrast, expression of PAD2 was restricted to the glandular epithelium and absent in the squamous epithelium in both normal mucosa and HPV-related lesions and did not seem to be involved in disease progression.

HPV vaccines demonstrate remarkable efficacy against infection and disease caused by vaccine-targeted types in clinical trials and real-world settings following the roll out of national vaccination programmes. In the absence of a defined correlate of protection, vaccine-induced antibodies are a useful proxy for vaccine-induced protective immunity. Vaccine-induced protection against type-specific cervical intraepithelial neoplasia (CIN) can be estimated in the context of vaccine trials but protection against disease irrespective of causal type is arguably a more useful metric from a cervical cancer elimination standpoint.

Here, Lehtinen et al., report data from 15 years of follow-up of 16- to 17-year-old participants enrolled in the bivalent (PATRICIA) and quadrivalent (FUTURE II) vaccine trials along with an unvaccinated cohort as a reference. These data provide assurance that early vaccination can reduce the risk of developing high-grade CIN, irrespective of causal type, and corroborate and extend observations made in other long term follow up studies from vaccine trials. The detection of vaccine-type antibodies 15+ years following initial vaccination supports the durability of vaccine-induced protection primarily mediated by antibody responses. Early vaccine studies used a three-dose regimen and while two-dose and one-dose regimens have more recently been adopted by national vaccine programmes, long-term follow up studies such as these are crucial to provide the benchmark evidence to support lifelong protection provided by the HPV vaccines.

Reduced vaccine effectiveness in immunocompromised individuals is of particular concern, particularly those infected with Human Immunodeficiency Virus and those undergoing immunosuppressive therapy following solid organ transplantation (SOT), with the latter tending to have poorer outcomes. The paper by Miyaji et al., presents results from an immunogenicity and safety trial comprising 18- to 45-year-old immunocompetent and SOT individuals receiving three doses of the quadrivalent vaccine. Vaccine-type seroconversion was 100% in the immunocompetent arm but ~65% in the SOT arm with antibody levels around 10-fold lower than those in the immunocompetent arm. Furthermore, the paper by dos Santos et al., describes a subset of these SOT individuals that having failed to seroconvert following three doses of vaccine were offered a fourth dose approximately 2 years later showing only an incremental improvement in seroconversion rate. High national vaccine coverage should ensure that future potential SOT patients will have been vaccinated as adolescents, but delivery of a booster vaccination prior to SOT could also be considered.

Olivera et al., present data on the impact of HPV infection on sperm quality parameters including higher reactive oxygen species and levels of necrosis compared to controls. The detection of HPV in semen has been associated with aberrant sperm parameters, reduced pregnancy rates and increased miscarriage rates, although the mechanisms have not been determined. Further work to understand the impact of HPV infection on reproductive health is warranted, although such risks should be reduced in countries with high rates of HPV vaccine coverage and particularly those that adopt gender neutral vaccination programmes.

We would like to thank the researchers who have chosen to submit their works to this Research Topic.

